# HtrA1 suppresses the growth of pancreatic cancer cells by modulating Notch-1 expression

**DOI:** 10.1590/1414-431X20187718

**Published:** 2018-11-23

**Authors:** Hao Cheng, Hao Zhu, Meng Cao, Chenglin Lu, Shanhua Bao, Yiming Pan

**Affiliations:** 1 Department of General Surgery, The Afflicted Drum Tower Hospital of Nanjing University Medical School, Nanjing, China; 2 Department of Gastroenterology, The Afflicted Drum Tower Hospital of Nanjing University Medical School, Nanjing, China

**Keywords:** Pancreatic cancer, Proliferation, HtrA1, Notch-1, Molecular target

## Abstract

Pancreatic cancer is well known to be the most deadly malignancy with the worst survival rate of all cancers. High temperature requirement factor A1 (HtrA1) plays an important role in cancer cell proliferation, migration, apoptosis, and differentiation. This study aimed to explore the function of HtrA1 in pancreatic cancer cell growth and its underlying mechanism. We found that the expression of HtrA1 was lower in pancreatic cancer tissue compared to the adjacent normal tissue. Consistently, HtrA1 levels were also decreased in two human pancreatic cancer cell lines, PANC-1 and BXPC-3. Moreover, enforced expression of HtrA1 inhibited cell viability and colony formation of PANC-1 and BXPC-3 cells. Overexpression of HtrA1 promoted apoptosis and suppressed migratory ability of tumor cells. On the contrary, siRNA-mediated knockdown of HtrA1 promoted the growth potential of pancreatic cancer cells. In addition, we found that up-regulation of HtrA1 reduced the expression of Notch-1 in pancreatic cancer cells. On the contrary, knockdown of HtrA1 increased the expression levels of Notch-1. Furthermore, overexpression of Notch-1 abolished the anti-proliferative effect of HtrA1 on pancreatic cancer cells. Taken together, our findings demonstrated that HtrA1 could inhibit pancreatic cancer cell growth via regulating Notch-1 expression, which implied that HtrA1 might be developed as a novel molecular target for pancreatic cancer therapy.

## Introduction

Pancreatic ductal adenocarcinoma, or pancreatic cancer, is one of the most malignant tumors with an estimated 277,000 new cases annually worldwide (1,2). Despite decades of continuous efforts, the five-year survival rate remains at the margin of 5% (3). The high mortality rate of pancreatic cancer is mainly due to the lack of early diagnosis and ineffective treatment strategies for advanced tumors. Thus, further investigation is critically required to provide novel therapeutic targets for successful treatment of pancreatic cancer (4).

High temperature requirement factor A1 (HtrA1), a member of the HtrA family of proteins, consists of a trypsin-like serine protease domain, a PDZ domain, an IGFBP/mac25-like domain, and a kazal-type inhibitor domain (5). HtrA1 has been shown to be involved in physiological and pathological processes such as osteoarthritis, preeclampsia, and leukoencephalopathy (6–8). In addition, accumulating evidence demonstrates that HtrA1 plays a role as a tumor suppressor in a variety of cancers, including breast cancer, gastric cancer, and hepatocellular carcinoma (9–12). Furthermore, it has been reported that the expression of HtrA1 is down-regulated in the progression and invasion of ovarian cancer, melanoma, lung cancer, and mesothelioma (9,13,14). Functional investigation reveals that up-regulation of HtrA1 could inhibit the proliferation, invasion and migration both *in vitro* and *in vivo* (13,15). However, the expression and functional relevance of HtrA1 in pancreatic cancer has not been investigated. Therefore, our current study aimed to explore the role of HtrA1 in the pathogenesis of pancreatic cancer as well as its potential underlying mechanism. Our findings could provide information on role of HtrA1 in the regulation of pancreatic cancer biological behaviors.

## Material and Methods

### Subjects

Twenty paired cancer tissues and non-tumorous tissues were collected from patients diagnosed with pancreatic cancer. Their clinical data were obtained during routine surgery at the department of Nanjing Medical University. There were eleven males (mean age 50.23±10.25 years) and nine females (mean age 49.53±11.06 years) in this study. None of them underwent preoperative chemotherapy or chemoradiotherapy. All patients signed an informed consent form before surgery, and the study protocol was approved by the ethics committee of Nanjing Medical University.

### Cell culture

Two pancreatic cancer cell lines, PANC-1 and BXPC-3, and a human pancreatic duct epithelial-like cell line hTERT-HPNE were purchased from ATCC (American Type Culture Collection, USA). Cells were cultured in Dulbecco's modified Eagle's medium (DMEM) (Thermo Fisher Scientific, USA) supplemented with 10% fetal calf serum (FCS, Sigma Aldrich, USA). All cell lines were cultured in an atmosphere of 5% CO_2_ and passaged when cell confluence reached 80%.

### Cell transfection

Pancreatic cancer cells were transfected with HtrA1-specific siRNA or negative control siRNA (Santa Cruz, USA) using Lipofectamine 2000 (Invitrogen, USA), according to the manufacturer's instructions. The transfection medium was replaced with normal culture medium 6 h after transfection. Subsequent experiments were performed 48 h after transfection and repeated in triplicate.

### CCK-8 assay

The proliferation of cells in each group was measured by CCK-8 assay. Briefly, cells at the density of 2.0×10^3^ cells/well were seeded onto a 96-well plate, and 100 µL fresh serum-free medium with 10 μL of CCK-8 solution was added to each well. Following incubation at 37°C, the medium was removed and absorbance was measured using a microplate reader (BioRad, USA).

### Colony formation assay

Cell clone formation was determined by colony formation assay. Briefly, pancreatic cancer cells at the density of 1.0×10^3^ cells/60 mm well were seeded in triplicate and incubated at 37°C to form clones. Upon clone formation, the cells were fixed with 4% paraformaldehyde and stained with crystal violet for 30 min. Subsequently, the number of cell clones on each plate was calculated.

### RNA extraction and real-time PCR

Total RNAs of pancreatic cancer tissues or cell lines was extracted using the RNAeasy Mini kit (Qiagen, USA) according to the manufacturer's instruction. Then, cDNA was reverse transcribed using a PrimeScrip™ RT reagent kit (Takara, Japan) according to the manufacturer's protocol. The real-time PCR parameters were set to determine the relative expression of indicated genes on ABI 7500 system (Applied Biosystems, USA). Beta-actin was applied as an internal control. Gene expression was measured with the 2^-ΔΔCt^ method. HtrA1: forward: 3′-TTGTTTCGCAAGCTTCCGTT-5′, reverse: 3′-ACGTGGGCATTTGTCACGAT-5′; Notch-1: forward: 3′-AATGTGGATGCCGCAGTTG-5′, reverse: 3′-ATCCGTGATGTCCCGGTTG-5′.

### Apoptosis assay

Cell apoptosis was determined with an annexin V/PI apoptosis detection kit according to the manufacturer's protocol (Invitrogen). Cells at a density of 1×10^6^cells/well were seeded onto a 6-well plate and 5 μL of annexin V-FITC and 5μL of PI were added into the cell suspension. Then, cell apoptosis was analyzed using a fluorescence-activated cell sorter (FACS, BD Biosciences, USA) according to the manufacturer's protocol.

### Migration assay

For cell migration assay, cells at the density of 10^4^ were seeded into the upper compartment (Millipore, USA) and allowed to migrate into the lower chamber. Cells remaining in the top chamber were removed and cells migrated to the lower membrane were stained and counted to evaluate their migratory ability.

### Western blot

The tissues or cells were lysed in lysis buffer (Invitrogen), and the proteins were subject to sodium dodecyl sulfate polyacrylamide gel electrophoresis (SDS-PAGE, Bio-Rad, USA) prior to being transferred to a polyvinylidene difluoride membrane. The membrane was incubated in phosphate buffered saline (PBS) with 5% nonfat dry milk. Subsequently, the membrane was incubated with primary antibodies (HtrA: ab38610, Notch-1: ab52627, diluted 1:1000 in PBS; Abcam, USA) followed by the appropriate peroxidase-coupled secondary antibody (#58802, diluted 1:2000 in PBS; Cell Signaling Technology, USA). Protein bands were detected by the enhanced chemiluminescence method.

### Statistical analysis

Data are reported as means±SD, and were analyzed by SPSS 17 software (SPSS, Inc., USA). Differences between groups were determined using Student's *t*-test or ANOVA. P values <0.05 were defined as statistically significant. All experiments were performed in triplicate.

## Results

### Dysregulated expression of HtrA1 in pancreatic cancer

**Figure 1.**
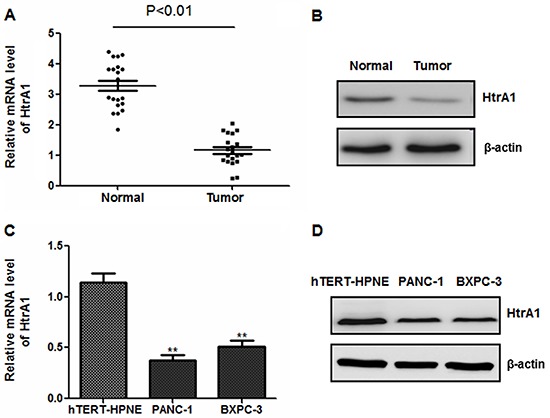
High temperature requirement factor A1 (HtrA1) expression was detected by real-time PCR and western blot in human pancreatic cancer tissue and adjacent normal tissue (*A* and *B*). Human pancreatic cancer cell lines including PANC-1 and BXPC-3 and a human pancreatic duct epithelial-like cell line hTERT-HPNE were subject to real-time PCR (*C*) and western blot (*D*) for detection of HtrA1 expression. Data are reported as means±SD. **P<0.01 (ANOVA).

Decreased expression of HtrA1 has been reported in several cancers, including gastric cancer, breast cancer, and melanoma (10,11,13). Hence, we examined whether HtrA1 levels were also reduced in pancreatic cancer. Real-time PCR and western blot analysis revealed that the mRNA and protein expression of HtrA1 was lower in pancreatic cancer tissue than the non-tumorous tissue (Figure 1A and B). These results implied that dysregulation of HtrA1 may play a role in the pathogenesis of pancreatic cancer. Subsequently, the expression of HtrA1 was detected in several human pancreatic cancer cell lines with the normal pancreatic epithelial cell line hTERT-HPNE used as control. Consequently, we found that the HtrA1 transcripts (Figure 1C) and protein expression were also reduced in the pancreatic cancer cell lines PANC-1 and BXPC-3 compared with the normal pancreatic epithelial cell line hTERT-HPNE (Figure 1D). Taken together, these data suggested that the expression levels of HtrA1 were lower in pancreatic cancer.

### Role of HtrA1 in inhibition of pancreatic cancer cell proliferation, apoptosis, and migration

**Figure 2.**
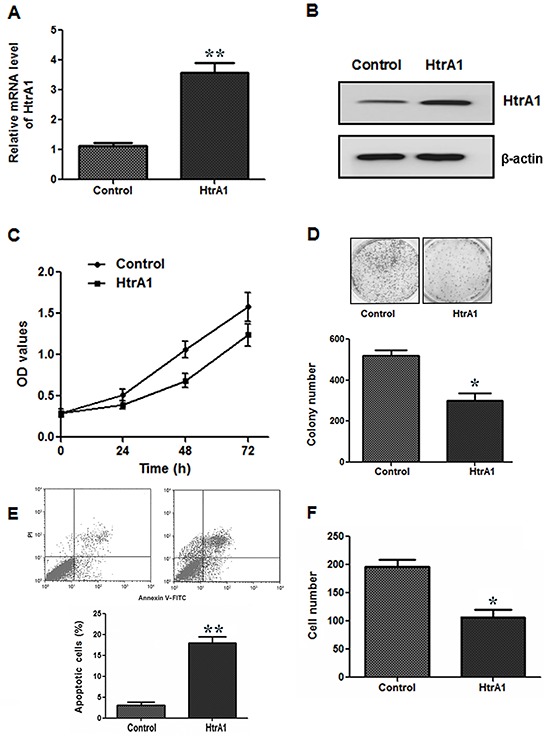
Role of up-regulated high temperature requirement factor A1 (HtrA1) on pancreatic cancer cell growth, apoptosis, and migration. After transfection with pcDNA3.1-HtrA1 plasmids, the expression of HtrA1 was measured by real-time PCR (*A*) and western blot (*B*). CCK-8 assay (*C*) and colony formation assay (*D*) were performed to detect the cell viability and colony formation of PANC-1 cells. Flow cytometry and Transwell assay were used to evaluate the apoptosis (*E*) and migration (*F*) of PANC-1 cells. Data are reported as means±SD. *P<0.05; **P<0.01 (*t*-test).

**Figure 3.**
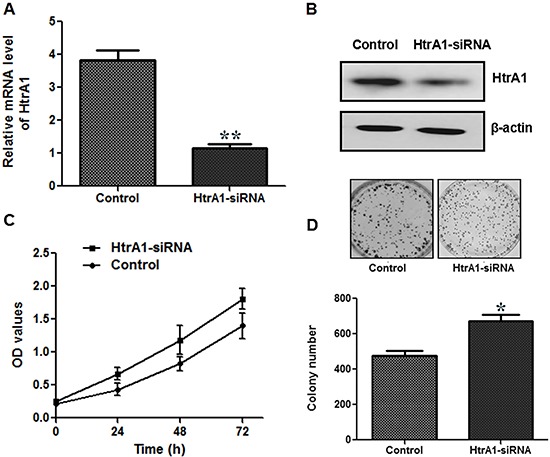
After transfection with high temperature requirement factor A1 (HtrA1)-specific siRNA, real-time PCR (*A*) and western blot (*B*) were performed to detect the expression of HtrA1 in PANC-1 cells. Cell viability and colony number were measured by CCK-8 assay (*C*) and colony formation assay (*D*), respectively. Data are reported as means±SD. *P<0.05; **P<0.01 (*t*-test).

The role of HtrA1 in the regulation of pancreatic cancer cell growth was further explored by CCK-8 assay. The pancreatic cancer PANC-1 cells were transfected with pcDNA3.1-HtrA1 plasmid to up-regulate the expression of HtrA1. After transfection, real-time PCR and western blot were performed to validate the successful up-regulated expression of HtrA1 in PANC-1 cells (Figure 2A and B). As a result, CCK-8 assay and colony formation assay showed that ectopic expression of HtrA1 significantly suppressed the growth ability and colony number of PANC-1 cells (Figure 2C and D). Moreover, we found that overexpression of HtrA1 promoted apoptosis and suppressed the migratory ability of tumor cells (Figure 2E and F). Furthermore, PANC-1 cells were transfected with HtrA1-specific siRNA for down-regulation of HtrA1. After transfection, we found that HtrA1 was decreased in PANC-1 cells both at the mRNA and protein levels (Figure 3A and B). Subsequently, cell viability and colony formation assay showed that the growth potential of PANC-1 cells was enhanced by down-regulation of HtrA1 (Figure 3C and D). Collectively, these findings demonstrated that HtrA1 served as a tumor suppressor in pancreatic cancer cells.

### HtrA1 was involved in regulating Notch-1 expression in pancreatic cancer cells

**Figure 4.**
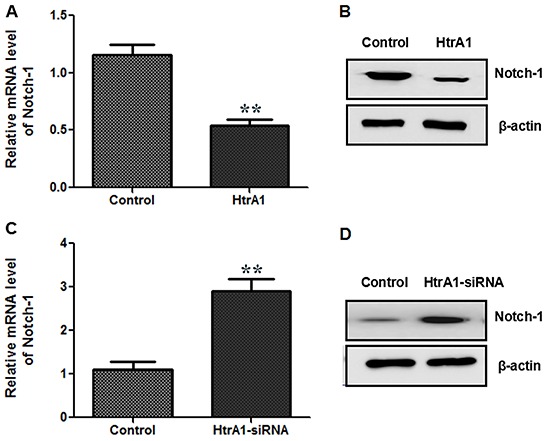
High temperature requirement factor A1 (HtrA1) regulated the expression of Notch-1 in pancreatic cancer cells. After transfection with pcDNA3.1-HtrA1 plasmids, the expression of Notch-1 was determined by real-time PCR (*A*) and western blot (*B*). Moreover, PANC-1 cells were transfected with HtrA1-siRNA, and then the Notch-1 transcripts (*C*) and protein levels (*D*) were measured by real-time PCR and western blot, respectively. Data are reported as means±SD. **P<0.01 (*t*-test).

We furthermore investigated the mechanism of HtrA1 in the proliferation of pancreatic cancer cells. It is well known that Notch signaling is frequently deregulated in human malignancies including pancreatic cancer. Thus, we investigated the role of HtrA1 in regulating Notch signaling in pancreatic cancer cells. Interestingly, our results showed that ectopic expression of HtrA1 inhibited the mRNA expression of Notch-1 in PANC-1 cells (Figure 4A). Moreover, western blot analysis suggested the down-regulated expression of Notch-1 in pcDNA3.1-HtrA1 plasmid-transfected pancreatic cancer cells (Figure 4B). On the contrary, transfection with HtrA1-specific siRNA significantly increased the Notch-1 transcripts in pancreatic cancer cells (Figure 4C). In addition, the protein expression of Notch-1 was obviously increased in PANC-1 cells upon transfection with HtrA1 siRNA (Figure 4D). These findings suggested that HtrA1 was negatively correlated with Notch-1 expression in pancreatic cancer cells.

**Figure 5.**
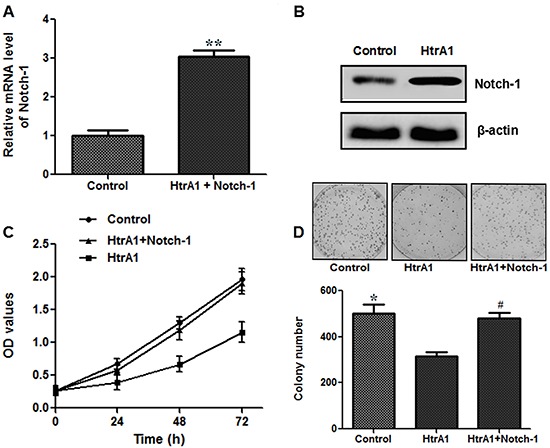
The pancreatic cancer PANC-1 cells over-expressing high temperature requirement factor A1 (HtrA1) were transfected with pcDNA3.1-Notch-1 plasmids. Then, real-time PCR and western blot were performed to determine the mRNA (*A*) and protein (*B*) levels of Notch-1, respectively. Cell viability and colony number were measured by CCK-8 assay (*C*) and colony formation assay (*D*). Data are reported as means±SD. *P<0.05; **P<0.01 compared to control; ^#^P<0.05 compared to cells over-expressing HtrA1.

Furthermore, we evaluated if HtrA1 played a role in the regulation of pancreatic cancer cells in a Notch-1-dependent manner. For this, pancreatic cancer cells were transfected with plasmids encoding Notch-1. The overexpression of Notch-1 was observed by real-time PCR (Figure 5A) and western blot (Figure 5B). Subsequently, cell viability was measured by CCK-8 and colony formation assay. We found that the suppressive function of HtrA1 on cell proliferation was abolished upon up-regulation of Notch-1 in pancreatic cancer cells (Figure 5C and D). Collectively, our findings revealed that HtrA1 played a critical role in the proliferation of pancreatic cancer cells mediated by Notch-1.

## Discussion

Pancreatic cancer has the worst prognosis among all major cancers and is the fourth most common cause of cancer-related death in the world (16). The lack of current effective therapies strongly encourages innovative investigation of the molecular mechanisms underlying pancreatic cancer (17). Our present study pointed out that HtrA1 could serve as a potential therapeutic target for pancreatic cancer treatment.

HtrA1, the first identified member of the HtrA family, consists of four distinct functional domains (5). Zumbrunn et al. first reported that the expression of HtrA1 was decreased in SV40-trasformed human fibroblasts (18). Later, it was shown that human HtrA1 was significantly elevated in the cartilage of osteoarthritis patients (19). Subsequently, several studies have demonstrated that the expression of HtrA1 is altered in various diseases including cancers (9,20). For example, Narklewicz et al. (21) observed a significant decrease of HtrA1 expression in ovarian tumors compared to the matched normal tissues. Another study revealed that the protein levels of HtrA1 were lower in gastric cancer tissue than those in normal gastric tissue (10). Functional investigation reveals that HtrA1 plays a critical role in cancer cell behaviors, including proliferation, migration, invasion, differentiation, and chemoresistance (10,12,22 [Bibr B23]). However, the expression profile and functional relevance of HtrA1 in pancreatic cancer has not been reported. In the present study, we found that the mRNA expression of HtrA1 was lower in pancreatic cancer tissue than in the adjacent normal tissue. Consistently, the expression levels of HtrA1 were decreased in pancreatic cancer cells compared with those in the normal pancreatic epithelial cells. These results demonstrated that HtrA1 was down-regulated in pancreatic cancer, suggesting a potential role of HtrA1 in the pathogenesis of this deadly disease.

It has been reported that overexpression of HtrA1 in several cancers could suppress cell growth, migration, and invasion, while HtrA1 knockdown in cancers induces resistance to conventional chemotherapeutics (10,12,22–24). To verify the biological role of HtrA1 in pancreatic cancer, we performed CCK-8 and colony formation assays to determine the growth potential of PANC-1 and BXPC-3 cells. As a result, we found that forced expression of f HtrA1 inhibited the growth ability of pancreatic cancer cells. Moreover, results showed that overexpression of HtrA1 promoted the apoptosis and suppressed the migratory ability of tumor cells. On the contrary, down-regulation of HtrA1 promoted cell viability and colony formation in PANC-1 and BXPC-3 cells. Therefore, our results pointed out that HtrA1 functioned as a tumor suppressor in pancreatic cancer cells.

Notch signaling plays crucial roles in cell growth, apoptosis, migration, and differentiation (25,26). Alterations in Notch signaling have been shown to be associated with tumorigenesis (27). Accumulating studies have reported the dysregulated expression of Notch-1 in several cancers, including pancreatic cancer (28,29). In addition, Zhang et al. (30) showed that paeoniflorin, a component of Chinese peony, inhibits the growth and invasion of breast cancer cells by regulating Notch-1 signaling pathway. In addition, down-regulation of Notch-1 suppresses cell growth and induces apoptosis in pancreatic cancer cells (31). In the current research, we found that enforced expression of HtrA1 suppressed Notch-1 expression in pancreatic cancer cells. In contrast, HtrA1-specific siRNA knockdown enhanced the expression levels of Notch-1 in pancreatic cancer cells. In addition, we found that overexpression of Notch-1 reversed the suppressive effect of HtrA1 on tumor cell growth, suggesting that the anti-proliferative ability of HtrA1 was dependent on Notch-1 in pancreatic cancer.

In conclusion, the present study showed that HtrA1 inhibited the proliferation of pancreatic cancer cells by modulating Notch-1 expression. Our findings demonstrated that HtrA1 could serve as a potential therapeutic target for the treatment of pancreatic cancer.
